# Supplementary material for: DNA metabarcoding and spatial modelling link diet diversification with distribution homogeneity in European bats
Source: Nat Commun. 2020 Mar 2;11:1154. doi: 10.1038/s41467-020-14961-2 (PMC7052159; doi:10.1038/s41467-020-14961-2)
Supplement: Supplementary file 3 — Reporting Summary [file 41467_2020_14961_MOESM3_ESM.pdf]

nature research

Corresponding author(s): Antton Alberdi

Last updated by author(s): Jan 20, 2020

Reporting Summary

Nature Research wishes to improve the reproducibility of the work that we publish. This form provides structure for consistency and transparency in reporting. For further information on Nature Research policies, see Authors & Referees and the Editorial Policy Checklist .

Statistics

For all statistical analyses, confirm that the following items are present in the figure legend, table legend, main text, or Methods section.

n/a	Confirmed	
☐	☒	The exact sample size (*n*) for each experimental group/condition, given as a discrete number and unit of measurement
☐	☒	A statement on whether measurements were taken from distinct samples or whether the same sample was measured repeatedly
☐	☒	The statistical test(s) used AND whether they are one- or two-sided<br>*Only common tests should be described solely by name; describe more complex techniques in the Methods section.*
☒	☐	A description of all covariates tested
☒	☐	A description of any assumptions or corrections, such as tests of normality and adjustment for multiple comparisons
☐	☒	A full description of the statistical parameters including central tendency (e.g. means) or other basic estimates (e.g. regression coefficient) AND variation (e.g. standard deviation) or associated estimates of uncertainty (e.g. confidence intervals)
☐	☒	For null hypothesis testing, the test statistic (e.g. *F*, *t*, *r*) with confidence intervals, effect sizes, degrees of freedom and *P* value noted<br>*Give P values as exact values whenever suitable.*
☒	☐	For Bayesian analysis, information on the choice of priors and Markov chain Monte Carlo settings
☐	☒	For hierarchical and complex designs, identification of the appropriate level for tests and full reporting of outcomes
☐	☒	Estimates of effect sizes (e.g. Cohen's *d*, Pearson's *r*), indicating how they were calculated

Our web collection on statistics for biologists contains articles on many of the points above.

Software and code

Policy information about availability of computer code

Data collection	No software was used for data collection.
Data analysis	For DNA metabarcoding bioinformatics: AdapterRemoval 2.2.2, DAMe 1.0, ncbi-blast 2.6.0+, R 3.2.0, Sumaclust 1.0.34, Clustal-omega/1.2.1, Beast/1.8.2, GraPhlan 0.9.7. For Species Distribution Modelling: biomod2 3.3-7.1, ENMTools 0.2. For statistics: R 3.2.0

For manuscripts utilizing custom algorithms or software that are central to the research but not yet described in published literature, software must be made available to editors/reviewers. We strongly encourage code deposition in a community repository (e.g. GitHub). See the Nature Research guidelines for submitting code & software for further information.

Data

Policy information about availability of data

All manuscripts must include a data availability statement. This statement should provide the following information, where applicable:

- Accession codes, unique identifiers, or web links for publicly available datasets
- A list of figures that have associated raw data
- A description of any restrictions on data availability

The datasets generated during and/or analysed during the current study are available in Zenodo with the following digital object identifier: 10.5281/zenodo.3610756 (ref. 63). The source data underlying Figs 1b-d, 2h-i, and 3a-d are provided as a Source Data file.

Field-specific reporting

Please select the one below that is the best fit for your research. If you are not sure, read the appropriate sections before making your selection.

☐ Life sciences ☐ Behavioural & social sciences ☒ Ecological, evolutionary & environmental sciences

October 2018

Ecological, evolutionary & environmental sciences study design

All studies must disclose on these points even when the disclosure is negative.

Study description	Nested hierarchy. Study unit: bat individual. Individuals (402) > Species (7).
Research sample	2-3 faecal droppings representing a snapshot of the trophic niche of 402 individual bats belonging to the species Miniopterus schreibersii (MSc), Myotis capaccinii (MCa), Myotis daubentonii (MDa), Myotis emarginatus (MEm), Myotis myotis (MMy), Rhinolophus euryale (REu) and Rhinolophus ferrumequinum (RFe).
Sampling strategy	Animals were trapped in roost entrances using harp-traps and/or mist-nets when returning from foraging (1am-7am), which ensured rapid defecation. Appropriateness of sample size was assessed in terms of rarefaction and extrapolation of captured diversity.
Data collection	Data for the Species Distribution Models was gathered from the online databases GBIF (www.gbif.org) and EUROBATS (https://www.eurobats.org/), from 33 journal publications, and unpublished records held by co-authors, as detailed in the Supplementary information.
Timing and spatial scale	Field work was performed between June and October in 2015, 2016 and 2017. These are the active periods of bats. Breeding season (May-early June) was avoided for conservation reasons.
Data exclusions	Samples with less than 5000 sequencing reads were excluded to avoid sequencing depth biases.
Reproducibility	Bioinformatic codes have been included in the Supplementary material and raw data will be deposited in a public repository.
Randomization	Molecular analyses were performed after randomly assigning samples to batches of 23 samples + 1 control using a custom R script.
Blinding	Blinding was not possible in this study, but to minimise biases, data collection and analysis of the trophic niche, spatial niche and ecological traits were performed by different research groups or researchers.

Did the study involve field work? ☒ Yes ☐ No

Field work, collection and transport

Field conditions	The conditions varied in each of the 40 samplings.
Location	Details of sampling locations are listed in Table S1.
Access and import/export	All animal captures, sample collection and transfer were conducted following the national legislation and in accordance of the specific capture permits.
Disturbance	Traps were set when animals returned from foraging, which enabled overcoming starvation issues. Due to ethical reasons, only 5 animals per species were sampled per site, and traps were removed when the objectives were achieved. All young animals, and pregnant and breeding females were released immediately.

Reporting for specific materials, systems and methods

We require information from authors about some types of materials, experimental systems and methods used in many studies. Here, indicate whether each material, system or method listed is relevant to your study. If you are not sure if a list item applies to your research, read the appropriate section before selecting a response.

Materials & experimental systems

n/a	Involved in the study	
☒	☐	Antibodies
☒	☐	Eukaryotic cell lines
☒	☐	Palaeontology
☐	☒	Animals and other organisms
☒	☐	Human research participants
☒	☐	Clinical data

Methods

n/a	Involved in the study	
☒	☐	ChIP-seq
☒	☐	Flow cytometry
☒	☐	MRI-based neuroimaging

Animals and other organisms

Policy information about studies involving animals; ARRIVE guidelines recommended for reporting animal research

Laboratory animals	n/a
Wild animals	Animals were trapped in roost entrances using harp-traps and/or mist-nets when returning from foraging (1am-7am), which ensured rapid defecation. To avoid sample cross-contamination, each bat was kept separately in a clean, single-use, UV-radiation sterilised cotton bag for 15-20 minutes, then identified, sexed and aged before releasing it into the cave.
Field-collected samples	Faecal pellets were collected from the bags and stored in 1.5 ml tubes filled with silica gel granules (Chameleon® C 1-3 mm, VWR) or absolute ethanol. Samples were kept dried and refrigerated (4-8 ºC) until they were transported to the laboratory, after which they were stored at -20 ºC.
Ethics oversight	All captures were authorized according to the laws of the countries where they were carried out. National and regional permits that authorised captures and sample collection: Bulgaria: Bulgarian Government 554/20.01.2014; Croatia: Ministarstvo Zastite Okolisa i Prirode UP/I-612-07/15-48/144; Greece: Operational Programme Environment & Sustainable Development (No permit needed for being a state survey); Italy: Italian Ministry of Environment 0005316/PNM 11 March 2016; Portugal: Instituto da Conservaçao de Natureza e das Florestas 452/2016/CAPT; Romania: Speleological Heritage Commission Nr. 34 / 14 ianuarie 2015; Serbia: Ministry of Agriculture and Environmental Protection of Serbia 353-01-1994/2014-17; Slovakia: Ministry of Environment of Slovakia 5050/2013-2.2; Spain: Junta de Andalucia 42521; Generalitat Valenciana 274/2016-VS; Gobierno de Aragón CSVIQ-7O2TA-26KAX-ZGREG; Arabako Foru Aldundia 15/104; Bizkaiko Foru Aldundia G13 1061; G13 1064; G13 1066; Ukraine: Ethics Commission of the V.N. Karazin Kharkiv National University 5.16, 01/02/2016; United Kingdom: Natural England 2018-36143-Sci-Sci, 2017-30137-SCI-SCI, 2017-28767-SCI-SCI, 2016-27156-SCI-SCI-3

Note that full information on the approval of the study protocol must also be provided in the manuscript.
